# Inappropriate Peak Inspiratory Flow Rate with Dry Powder Inhaler in Chronic Obstructive Pulmonary Disease

**DOI:** 10.1038/s41598-020-64235-6

**Published:** 2020-04-29

**Authors:** Shih-Yu Chen, Chun-Kai Huang, Hui-Chuan Peng, Chong-Jen Yu, Jung-Yien Chien

**Affiliations:** 1 Department of Internal Medicine, National Taiwan University Hospital, College of Medicine, National Taiwan University, Taipei, Taiwan; 2 Institute of Epidemiology and Preventive Medicine, College of Public Health, National Taiwan University, Taipei, Taiwan; 3 Department of Nursing, National Taiwan University Hospital, College of Medicine, National Taiwan University, Taipei, Taiwan

**Keywords:** Chronic obstructive pulmonary disease, Respiratory signs and symptoms

## Abstract

Optimal peak inspiratory flow rate (PIFR) is crucial for optimizing dry powder inhaler (DPI) effectiveness for chronic obstructive pulmonary disease (COPD). This study provide an insight that there was a substantial proportion of improper PIFRs (not only insufficient but also excessive) among COPD patients using DPIs. We enrolled 138 COPD patients from a medical center in Taiwan and measured PIFRs against different internal resistances of DPIs. Proportion of excessive, optimal, suboptimal, and insufficient PIFRs were 2%, 54%, 41%, 3%, respectively, against medium-high resistance; 2%, 77%, 20%, 1%, respectively, against medium resistance; 27%, 63%, 9%, 1%, respectively, against medium-low resistance; and 42%, 57%, 1%, 0%, respectively, against low resistance (p < 0.01). Although most PIFRs against medium-high (54%), medium (77%), medium-low (63%) and low (57%) resistance were optimal, a substantial proportion of PIFRs against low resistance were excessive (42%, p < 0.01), irrespective of age, body-mass index, dyspnea severity score, and COPD severity. Insufficient PIFRs were infrequent, but suboptimal/insufficient PIFRs were most prevalent in patients older than 75 years than in younger patients (36% vs. 56%, p = 0.036) against medium-high resistance. Regularly monitoring PIFRs against the specific resistance of the DPIs and instructing patients to employ a proper inspiration effort may help to optimize the effects of DPIs.

## Introduction

Dry powder inhalers (DPIs), a breath-actuated inhalation systems, required patients to generate sufficient inspiratory flow and turbulence in the device to disaggregate the powder into fine particles^1,2^. Thus, it is generally advised to inhale with a forced inspiratory maneuver to generate adequate peak inspiratory flow rate (PIFR) to overcome the internal resistance of the devices^1–5^. The PIFR is impacted by several factors, such as sex, age, height, the internal resistance of DPIs, inhalation effort, pulmonary function, and even the period following acute exacerbation due to chronic obstructive pulmonary disease (COPD)^6–8^. It is generally suggested that PIFR less than 30 L/min is insufficient and the suggested optimal PIFR is at least 60 L/min for Turbuhaler, Ellipta and Accuhaler, and 50 L/min for Breezhaler^5,6^. However, compared to optimal PIFR, excessive PIFR also lead to more oropharyngeal deposition and less lung deposition and a PIFR more than 90 L/min was considered excessive^9,10^.

Different DPIs have different internal resistances, which can be categorized to medium-high, medium, medium-low and low internal resistances^11^. We aim to investigate the prevalence of improper PIFRs and the influencing factors against different internal resistances of DPIs, among COPD patients with varying disease severity.

## Methods

Adult patients with stable COPD, who receiving medical treatment in our outpatient clinics without acute exacerbation during previous 3 months, were recruited from National Taiwan University Hospital from May 2017 to February 2019. The patients were diagnosed according to the GOLD criteria defined by <70% post-bronchodilator forced expiratory volume in one second (FEV1) to forced vital capacity (FVC) ratio (FEV1/FVC ratio). Data on patients’ demographics, results of pulmonary function tests, smoking status, dyspnea severity classified by scores of modified medical research council (mMRC) and COPD assessment test (CAT) were recorded. According to GOLD guideline, we classified COPD patients into GOLD Group ABCD according to frequency of exacerbation and mMRC or CAT scoring system with the greater score. PIFRs were measured against four-degrees of internal resistances (low, medium-low, medium, and medium-high) using the In-Check Dial G16 (Clement-Clarke International Ltd, Harlow, UK), a handheld inspiratory flow device simulates different internal resistances of DPIs^11^. For low resistance devices, measured PIFRs were classified to excessive (≥100 L/min), optimal (50–99 L/min), suboptimal (30–49 L/min), and insufficient (<30 L/min)^12^, and for those other than low resistance devices, measured PIFRs were classified into four categories, excessive (≥90 L/min), optimal (60–89 L/min), suboptimal (30–59 L/min), and insufficient (<30 L/min)^5,6,10^. The institution board of National Taiwan University Hospital (201905058RINB) approved this study and written informed consent was waived by the ethics committee due to the retrospective nature of the study. All methods were carried out in accordance with relevant guidelines and regulations.

### Statistical analysis

Categorical variables were compared using a chi-square test or Fisher’s exact test, as appropriate. Differences in continuous variables were analyzed using the Mann-Whitney U test or ANOVA. The data are presented as numbers (percentages), median (range), mean ± standard deviation, and p = 0.05 is considered a statistically significant difference. Linear regression analysis was used to investigate the relationship between PIFR and FEV1, FVC, age and body height. The statistical analyses were performed using STATA version 14 software (StataCorp LLC, TX).

## Results

A total of 138 patients with stable COPD underwent PIFR measurement during the study. The median age was 72 (37–91) years, and most participants were men (131, 94.9%). The median height was 164 (146–180) cm while the median weight was 63 (40–102) kg. Mean forced expiratory volume in one second (FEV1) was 1.53 ± 0.49 L, and the mean percentage of predicted value was 70.9 ± 21.8%. Table 1 shows the demographic parameters.

**Table 1:** Characteristics of patients with chronic obstructive pulmonary disease.

Variable	All	GOLD Group				p Value
		Group A(n = 36)	Group B(n = 75)	Group C(n = 7)	Group D(n = 20)	
Age in years	72 (37–91)	71 (37–87)	72 (54–85)	79 (65–82)	73 (56–87)	0.406
Male	131 (95)	32 (89)	73 (97)	6 (86)	20 (100)	0.099
Weight, kg	63 (40–102)	63.2 (43–87)	62.9 (40–102)	56 (49–71)	66 (52–81)	0.319
Height, cm	164 (146–180)	162 (147–180)	162.8 (146–180)	165 (158–170)	164.5 (158–172)	0.567
Body mass index, kg/m^2^	23.2 (16–36.1)	22.85 (19.4–29.1)	23.7 (16–36.1)	20.6 (19.1–21.6)	24 (19.3–28.6)	0.289
FEV1, L	1.46 (0.45–3.25)	1.79 (0.75–3.25)	1.35 (0.45–2.54)	1.46 (0.85–2.32)	1.36 (0.83–2.44)	0.009
FEV1, % predicted	71.8 (22.3–131.1)	80 (34.5–130.9)	69.2 (22.3–131.1)	75.7 (54.9–89.8)	63.6 (32.3–124.4)	0.014
Smoking status						0.576
Current smoker	28 (20)	10 (28)	14 (19)	1 (14)	3 (15)	
Former smoker	74 (54)	18 (50)	44 (59)	3 (43)	9 (45)	
Never smoker	19 (14)	3 (8)	10 (13)	2 (29)	4 (20)	
mMRC dyspnea score						<0.001
Grade 0	4 (3)	3 (8)	1 (1)	0	0	
Grade 1	43 (31)	33 (92)	3 (4)	7 (100)	0	
Grade 2	67 (49)	0	56 (75)	0	11 (55)	
Grade 3	24 (17)	0	15 (20)	0	9 (45)	
Grade 4	0	0	0	0	0	
CAT score						<0.001
≤10	106 (77)	36 (100)	51 (68)	7 (100)	12 (60)	
11–20	29 (21)	0	23 (31)	0	6 (30)	
21–30	3 (2)	0	1 (1)	0	2 (10)	
31–40	0	0	0	0	0	
**Comorbidity**						
Cerebrovascular accident or neuromuscular disease	12 (9)	1 (3)	10 (13)	1 (14)	0	0.120
Head and neck tumor	5 (4)	2 (6)	3 (4)	0	0	0.698
Cardiovascular diseases	50 (36)	7 (19)	31 (41)	2 (29)	10 (50)	0.070
Asthma	13 (9)	2 (6)	7 (9)	1 (14)	3 (15)	0.670
Hypertension	65 (47)	13 (36)	40 (53)	0	12 (60)	0.015
Diabetes mellitus	36 (26)	8 (22)	17 (23)	0	4 (20)	0.566

Data presented as n (%) or median (range).

CAT, Chronic obstructive pulmonary disease assessment test; FEV1, forced expiratory volume in one second; GOLD: Global Initiative for Chronic Obstructive; mMRC, modified medical research council.

**Figure 1:**
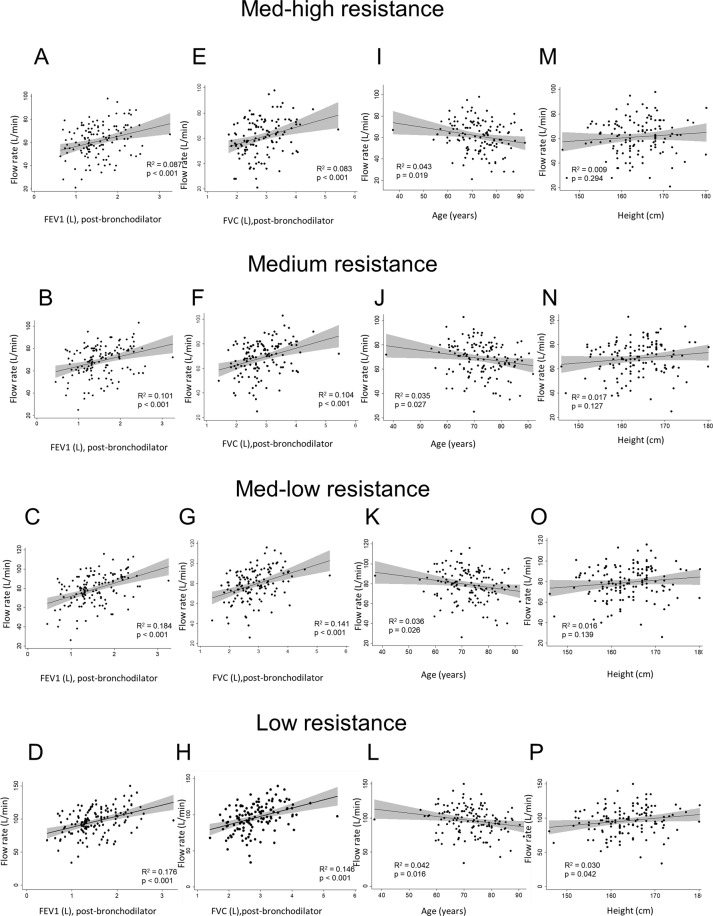
Scatter plot and regression line between peak inspiratory flow rate and forced expiratory volume in one second (FEV1, Panel A–D), percent predicted value of forced vital capacity (FVC%, Panel E–H), age (Panel I–L) and body height (Panel M–P) against different simulated internal resistances of dry powder devices.

**Figure 2:**
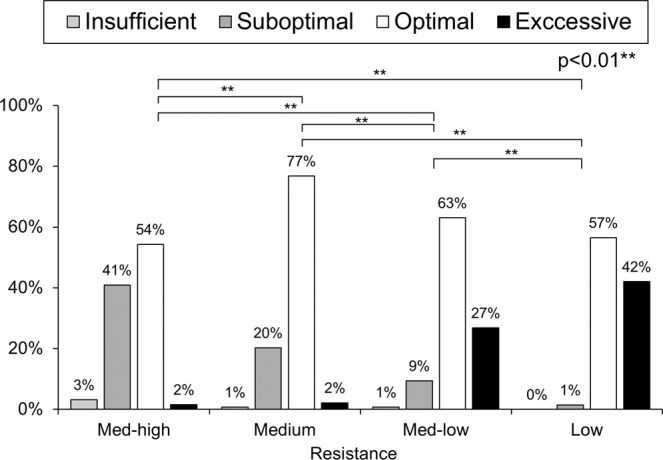
Prevalence of excessive, optimal, suboptimal, and insufficient peak inspiratory flow rates measured against different resistances (p < 0.01). ** represents p < 0.01 between each resistance.

**Figure 3:**
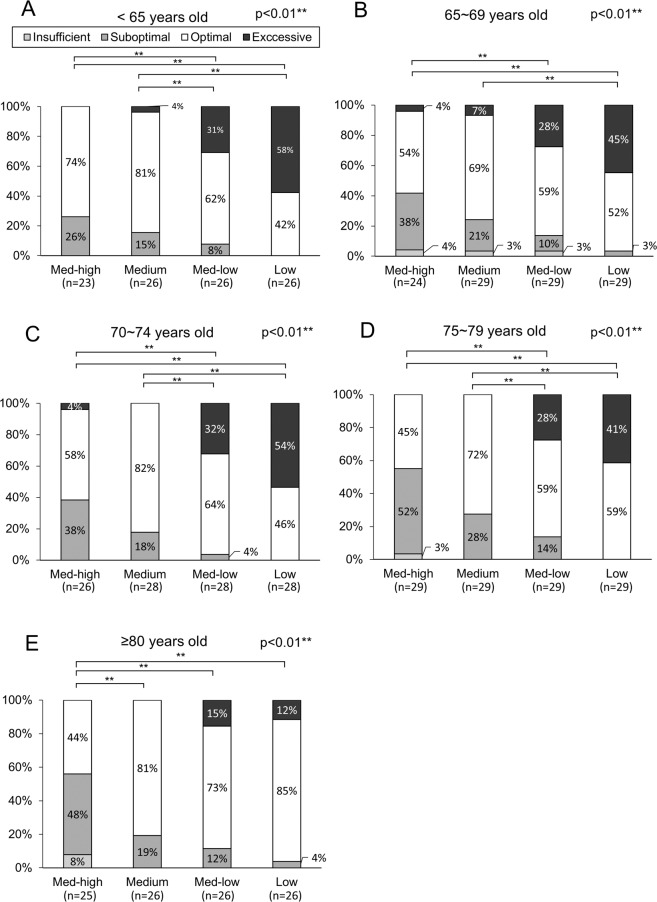
Percentage of excessive, optimal, suboptimal and insufficient peak inspiratory flow rates measured against different resistances among patients <65 (Panel A, p < 0.01), 65–69 (Panel B, p < 0.01), 70–74 (Panel C, p < 0.01), 75–79 (Panel D, p < 0.01) and ≥80 years of age (Panel E, p < 0.01). * represents p < 0.05, ** represents p < 0.01.

**Figure 4:**
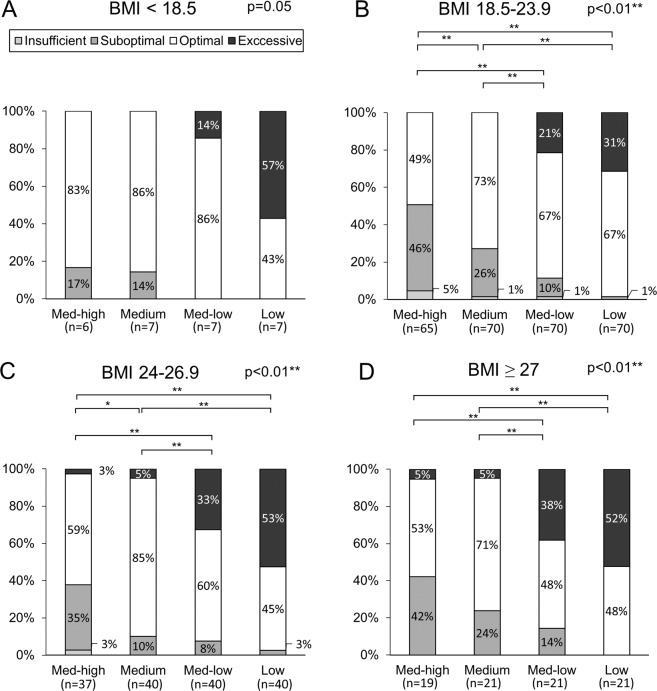
Percentage of excessive, optimal, suboptimal and insufficient peak inspiratory flow rates measured against different resistances among patients with body mass index <18.5 (Panel A, p < 0.01), 18.5–23.9 (Panel B, p < 0.01), 24–26.9 (Panel C, p < 0.01) and ≥ 27 kg/m^2^ (Panel D, p < 0.01). * represents p < 0.05; ** represents p < 0.01.

**Figure 5:**
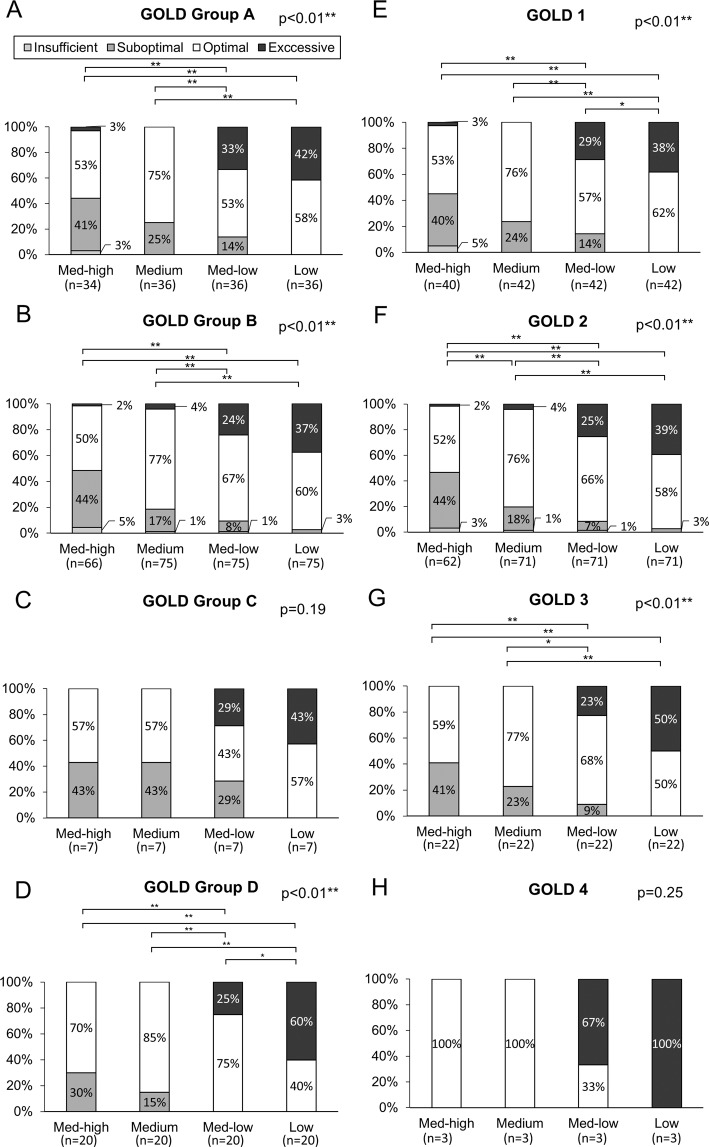
Percentage of excessive, optimal, suboptimal and insufficient peak inspiratory flow rates measured against different resistances among patients with Global Initiative for Chronic Obstructive Lung Disease (GOLD) group A (Panel A, p < 0.01), group B (Panel B, p < 0.01), group C (Panel C, p = 0.22), group D (Panel D, p < 0.01) and GOLD stage 1 (Panel E, p < 0.01), stage 2 (Panel F, p < 0.01), stage 3 (Panel G, p < 0.01) and stage 4 (Panel H, p = 0.045). * represents p < 0.05; ** represents p < 0.01.

The median PIFR was 63 (21–98) L/min against medium-high, 71 (25–103) L/min against medium, 80 (26–116) L/min against medium-low, and 97 (34–150) L/min against low resistances. Measured PIFRs positively correlated with FEV1 and forced vital capacity (FVC) but negatively correlated with age and didn’t correlate with body height (Fig. 1). The percentage of insufficient, suboptimal, optimal and excessive PIFRs against low resistance were 0% (n = 0), 1% (n = 2), 57% (n = 78), 42% (n = 58), respectively, against medium-low resistance were 1% (n = 1), 9% (n = 13), 63% (n = 87), 27% (n = 37), respectively, and against medium resistance were 1% (n = 1), 20% (n = 28), 77% (n = 106), 2% (n = 3), respectively (p < 0.01). Among 127 patients, PIFRs measured against medium-high resistance of DPIs, 4 (3%) were insufficient, 52 (41%) were suboptimal, 69 (54%) were optimal, and 2 (2%) were excessive, respectively (Fig. 2, p < 0.01). Further subgroup analysis in Table 2 and Figs. 3–5 show that regardless of age groups, gender, body mass index (BMI), severity of dyspnea by mMRC score, GOLD stages or GOLD groups, there were more optimal PIFRs as measured against medium internal resistance, while there was a majority of excessive PIFRs as measured against low resistance and more suboptimal PIFRs as measured against medium-high resistance. Figure 3 also shows that patients >75 years have a higher prevalence of suboptimal or insufficient PIFRs than younger patients (36% vs. 56%, p = 0.036) when measured against DPIs with medium-high internal resistance.

**Table 2:** Forced peak inspiratory flow rates against different simulated internal resistances of dry powder inhalers.

Variable	Internal resistance			
	Med-high	Medium	Med-low	Low
**Age, years**				
<65	68 (36–85)	75.5 (44–95)	84 (47–103)	103.5 (62–128)
65–70	63.5 (21–95)	72 (25–103)	80 (26–113)	100 (34–150)
70–75	64.5 (40–98)	74.5 (46–90)	86 (53–116)	103.5 (66–128)
75–80	57 (28–85)	70 (38–86)	75 (46–98)	93 (58–135)
≥80	58 (28–82)	65.5 (35–83)	76.5 (38–95)	91.5 (43–115)
p value	0.126	0.204	0.188	0.124
**Body mass index, kg/m**^**2**^				
<18.5	70.5 (52–79)	76 (56–80)	86 (69–95)	104 (78–112)
18.5–24	59 (21–88)	66.5 (25–90)	75 (26–101)	95 (34–150)
24–27	66 (28–95)	72 (35–103)	82.5 (38–113)	102 (43–140)
≥27	62 (34–98)	74 (42–95)	86 (51–116)	103 (61–122)
p Value	0.283	0.068	0.036	0.300
**Sex**				
Man	63 (21–98)	71 (25–103)	81 (26–116)	98 (34–150)
Woman	56 (28–74)	60 (40–75)	73 (46–88)	89 (64–110)
p Value	0.171	0.04	0.077	0.182
**mMRC**				
Grade 0	46 (28–62)	51.5 (40–76)	57.5 (46–91)	70 (64–113)
Grade 1	64 (28–98)	72 (38–103)	84 (40–116)	97 (52–140)
Grade 2	60.5 (21–95)	70 (25–95)	78 (26–110)	97 (34–135)
Grade 3	63 (34–88)	70.5 (38–90)	82 (46–103)	102.5 (58–150)
p Value	0.138	0.140	0.157	0.275
**COPD GOLD stage**				
GOLD 1	62.5 (28–98)	71.5 (38–90)	82.5 (40–116)	96.5 (52–135)
GOLD 2	61.5 (21–95)	70 (25–103)	78 (26–113)	97 (34–140)
GOLD 3	62.5 (34–85)	67.5 (38–86)	79.5 (46–103)	101 (58–122)
GOLD 4	70 (68–88)	86 (74–90)	98 (86–101)	122 (103–150)
p Value	0.348	0.184	0.378	0.083
**COPD GOLD group**				
Group A	62.5 (28–98)	72 (40–90)	85 (46–116)	97 (64–135)
Group B	61.5 (21–95)	70 (25–103)	78 (26–113)	97 (34–140)
Group C	60 (34–84)	65 (38–86)	70 (46–102)	91 (58–122)
Group D	63.5 (40–88)	71 (44–90)	82 (61–103)	103 (68–150)
p Value	0.758	0.736	0.638	0.400

Data presented as median (range).

GOLD: Global Initiative for Chronic Obstructive; mMRC, modified medical research council.

## Discussion

We investigated the PIFRs against different internal resistances of DPIs among stable COPD patients and found correlations between PIFRs and FEV1, FVC, and age. Moreover, among stable COPD patients, we found that more PIFRs measured against medium resistance were optimal, more PIFRs against medium-high resistance were suboptimal, while a majority of PIFRs against low resistance were excessive.

Forced inspiration could provide faster acceleration rates, which increases the deaglommeration of particles before the dose leaves the device. Therefore, the guideline of inhalation therapies suggests “instruct the patient to inhale forcefully from the beginning”^1^. This forced inspiration method provides a simple and precise instruction to the patients. This method is easy to remember, readily found in most prescribing information for DPIs and allows patients to perform the inhalation consistently in daily life^5^. More importantly, without the maximum inhalation effort, the relationship between the flow rate and the pressure drop will be inconsistent^4^. A PIFR between 60 and 90 L/min was suggested as optimal by several previous studies^13–15^, and 30 L/min is considered the minimum effective PIFR, making the range of 30–60 L/min a debatable area^6,10,11,16^. Thus, in this study, this flow range of 30–60 L/min was classified as suboptimal while those less than 30 L/min as insufficient. Pavkov *et al*. studied 26 patients using Breezhaler, a low resistance device and found that a consistent fine particle mass can be achieved at rate of 50–100 L/min. In this way, the flow range of 50–100 L/min was classified as optimal while 30–49 L/min as suboptimal for low resistance devices^12^. High prevalence of suboptimal PIFRs were reported in previous studies which accounts for approximately 20–78% of studied population^5,13,17–21^. Similarly, our study found the proportion of suboptimal or insufficient PIFRs range from 3–44% among stable COPD patients with different severity.

However, although most studies emphasized on suboptimal PIFRs as a major problem of inappropriate DPI usage, we further found a substantial proportion of PIFRs were excessive when measured against medium-low and low resistance (27% and 42%, respectively). Excessive inspiratory flow rates also have negative impact on drug deposition in respiratory tract^2^. Usmani *et al*. used inhaled technetium-99m-labeled monodisperse albuterol aerosols and compared the respiratory deposition at slow and fast inspiratory flow rates, and found that faster inspiratory flows yield more oropharyngeal and central lung deposition (regardless of particle size), decreased total lung deposition, and lesser clinical effectiveness^9^. We found that patients tend to have excessive PIFRs as measured against medium-low or low resistance, while suboptimal or insufficient PIFR values resulted when measured against medium-high resistance. This remained true when patients were subdivided by BMI, age, COPD group or COPD stage, emphasizing that the prevalence of excessive or suboptimal peak inspiratory flow rates could be highly correlated with an internal resistance of DPIs *per se*.

Several pulmonary function parameters were also found to have association with PIFRs. Mahler *et al*. measured PIFR against medium-low resistance in COPD GOLD stage 3 and 4 patients (mean FEV1 of 0.92 ± 0.26 L) and found that PIFRs are associated with FVC and inspiratory capacity^19^. Duates *et al*. demonstrated a significant correlation between PIFRs and severity of air trapping, represented by the ratio of residual volume over total lung capacity (RV/TLC)^22^. We found values of PIFRs correlated positively with FEV1 and FVC. This finding was different from Janssens *et al*. where PIFRs were not statistically correlated with FEV1. This possibly is because they measured PIFRs against zero resistance and the smaller study population (26 COPD patients) in their study^13^.

Similar to previous studies^13,18–20^, we found a negative correlation between age and PIFRs (p < 0.05). Furthermore, as measured against medium-high resistance, patients more than 75 years have a higher prevalence of suboptimal or insufficient PIFR (36% vs. 56%, p = 0.036). In contrast, the prevalence of suboptimal or insufficient PIFRs did not significantly correlate with age among medium, medium-low, and low resistance of DPIs. It was similar to the study conducted by Kawamatawong *et al*. that older patients had a higher proportion of suboptimal PIFR breathing against Turbuhaler than Accuhaler (19.3% vs 9.3%)^18^.

There are some limitations to our study. First, our COPD population is predominantly male, which may not be representative of the spectrum of the COPD population worldwide, although our papulation was similar to a random cross-sectional national survey in Taiwan which showed males accounted for 78.9% of the COPD population^23^. However, this male predominant papulation could lead to female gender and short stature, two important predictors of reduced PIFRs in the previous studies, did not have significant roles in this study^17,19,20,22,24^. Second, as patients may not exert maximal inspiratory effort every time in daily life, there may be a difference between in-office evaluation and daily practice at home. The very small number of participants with BMI < 18.5 (n = 7), GOLD stage 4 (n = 3) and mMRC grade 0 (n = 4) substantially led to great variations and pseudo-higher PIFRs in patients with BMI < 18.5 and GOLD stage 4 and pseudo-lower PIFRs in those with mMRC grade 0 (all the differences were not statistically significant). Therefore, there was great limitation and the interpretation of those findings should be very cautious. Fourth, this is a cross-sectional study and all patients had received effective treatment and pulmonary rehabilitation program without inspiratory muscle training for a period of time. A further prospective study design was needed in the future to address the effects of treatment and pulmonary rehabilitation on the PIFR. Finally, our study population is mainly stable COPD patients without acute exacerbation in previous 3 months. The result cannot be applied to those experiencing acute exacerbation of chronic obstructive disease or who just recover from acute illness.

Our study identified that PIFRs are correlated with FEV1, FVC, and age. We also revealed a substantial proportion of improper PIFRs (excessive and suboptimal) as measured against different resistances of DPIs, but insufficient PIFRs were infrequent (<5%). The highest probability of optimal PIFR was measured against medium resistance. The lower the internal resistance, the higher the probability of excessive PIFR was noted, and suboptimal PIFRs were more often noticed when measured against higher internal resistance. Both excessive and suboptimal PIFRs may negatively impact drug deposition, therefore, regularly monitoring PIFR against the resistance of specific DPI and instructing patients to employ a proper inspiration effort may help to optimize the effects of DPIs.
